# X‐Ray Visible Protein Scaffolds by Bulk Iodination

**DOI:** 10.1002/advs.202306246

**Published:** 2023-12-25

**Authors:** Carlos Flechas Becerra, Lady V. Barrios Silva, Ebtehal Ahmed, Joseph C. Bear, Zhiping Feng, David Y.S. Chau, Samuel G. Parker, Steve Halligan, Mark F. Lythgoe, Daniel J. Stuckey, P. Stephen Patrick

**Affiliations:** ^1^ Centre for Advanced Biomedical Imaging Division of Medicine University College London Paul O'Gorman Building, 72 Huntley Street London WC1E 6DD UK; ^2^ Division of Biomaterials and Tissue Engineering Eastman Dental Institute University College London Royal Free Hospital Rowland Hill Street London NW3 2PF UK; ^3^ School of Life Science Pharmacy & Chemistry Kingston University Penrhyn Road Kingston upon Thames KT1 2EE UK; ^4^ Centre for Medical Imaging, Division of Medicine University College London UCL Charles Bell House, 43–45 Foley Street London W1W 7TS UK

**Keywords:** biomaterials, collagen, hernia, Iodine, silk, tissue engineering, tyrosine, X‐ray

## Abstract

Protein‐based biomaterial use is expanding within medicine, together with the demand to visualize their placement and behavior in vivo. However, current medical imaging techniques struggle to differentiate between protein‐based implants and surrounding tissue. Here a fast, simple, and translational solution for tracking transplanted protein‐based scaffolds is presented using X‐ray CT–facilitating long‐term, non‐invasive, and high‐resolution imaging. X‐ray visible scaffolds are engineered by selectively iodinating tyrosine residues under mild conditions using readily available reagents. To illustrate translatability, a clinically approved hernia repair mesh (based on decellularized porcine dermis) is labeled, preserving morphological and mechanical properties. In a mouse model of mesh implantation, implants retain marked X‐ray contrast up to 3 months, together with an unchanged degradation rate and inflammatory response. The technique's compatibility is demonstrated with a range of therapeutically relevant protein formats including bovine, porcine, and jellyfish collagen, as well as silk sutures, enabling a wide range of surgical and regenerative medicine uses. This solution tackles the challenge of visualizing implanted protein‐based biomaterials, which conventional imaging methods fail to differentiate from endogenous tissue. This will address previously unanswered questions regarding the accuracy of implantation, degradation rate, migration, and structural integrity, thereby accelerating optimization and safe translation of therapeutic biomaterials.

## Graphical abstract

Selective iodination of tyrosine residues provides a fast, robust, and cost‐effective route to engineering X‐ray visible protein scaffolds. Using one of the most widely used surgical biomaterials – collagen‐based hernia meshes, materials are visible up to 3 months post‐implantation in vivo with clinically‐relevant X‐ray doses. This will address previously unanswered questions on implantation accuracy, degradation rate, migration, and structural integrity.

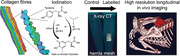

## 1 Introduction

The use of protein‐based biomaterials is widespread in surgery and regenerative medicine.^[^ ^1^, ^2^ ^]^ Among these collagen is predominant,^[^ ^3^ ^]^ with established and emerging clinical applications in repairing urogenital damage, skin wounds, bone defects, hernia, cardio‐vascular disease, and in surgical reconstruction.^[^ ^4^, ^5^, ^6^, ^7^, ^8^, ^9^ ^]^ While the endogenous variety and complexity of decellularized donor tissues (dermis, aortic valves,^[^ ^10^ ^]^ lung,^[^ ^11^ ^]^ heart ^[^ ^12^, ^13^ ^]^ etc.) allow close matching to the needs of a given therapy, this can also be achieved by synthetic bottom‐up engineering to allow the complete design of the material.^[^ ^3^, ^14^ ^]^ Advanced manufacturing techniques including 3D printing, electrospinning, moulding, microfluidic encapsulation, acoustic cell‐patterning, crosslinking, and composite and modular assembly allow artificial control of degradation rate, structure, and patterning across multiple length scales.^[^ ^5^, ^15^, ^16^, ^17^, ^18^, ^19^ ^]^ Through their combination with cell therapy, growth factors, and nanoparticles, unmet needs in spinal cord damage,^[^ ^20^ ^]^ diabetes,^[^ ^21^ ^]^ cartilage and connective tissue defects, tooth,^[^ ^22^ ^]^ and heart regeneration,^[^ ^17^ ^]^ are now being approached. Beyond collagen, fibrous proteins including silk, elastin and keratin are growing in their use as biomaterials, further expanding the range of material and biological properties available, and the potential for novel therapeutic applications.^[^ ^5^, ^19^ ^]^

Yet in order for emerging biomaterial therapies to reach maturity, it is crucial to validate their behavior and safety post‐implantation.^[^ ^23^, ^24^, ^25^ ^]^ However, this task remains challenging due to the inherent resemblance between protein‐based implants and native tissue, with both generating similar signals on commonly used medical imaging modalities such as Magnetic Resonance Imaging (MRI), X‐ray Computed Tomography (CT), and ultrasound. Consequently, detection of critical events such as successful delivery and integration, mis‐delivery, mechanical failure, or detachment proves unachievable in both preclinical models and patient populations, primarily due to the limited availability of imageable scaffolds. Though both X‐ray visible collagen and silk materials have been previously developed, these have suffered either from a reduction in material strength due to labeling,^[^ ^26^ ^]^ or the low practicality of stably and homogenously incorporating nanoparticle‐based contrast agents into pre‐formed scaffolds such as decellularized tissue.^[^ ^27^ ^]^ Though various chemistries are compatible with whole collagen scaffolds including aldehyde or carbodiimide‐based reactions for crosslinking,^[^ ^3^, ^6^ ^]^ conjugation of cell attachment sites, and introduction of biochemically active groups,^[^ ^28^, ^29^ ^]^ there has been little focus on enabling non‐invasive in vivo imaging.

To address this, we developed a fast, simple, and selective labeling approach to produce radiopaque, X‐ray visible protein‐based biomaterials that retain their material properties. Here we leveraged two iodination reactions that have been historically employed to bind radioactive iodine isotopes (^123^I, ^124^I, or ^131^I) to tyrosine residues in soluble proteins such as antibodies.^[^ ^30^ ^]^ To our knowledge, this is the first time either of these reactions have been applied to produce X‐ray‐based contrast or adapted for compatibility with macroscopic protein scaffolds with or without a stable, non‐radioactive iodine isotope.

First, we tested a modified version of the potassium tri‐iodide reaction,^[^ ^31^ ^]^ popularized by Pressman and colleagues in the late 1940s.^[^ ^32^, ^33^, ^34^ ^]^ To adapt this for non‐radio‐labeling purposes, we used a preparation of Lugol's iodine as a source of potassium tri‐iodide (KI_3_) at pH 7.4. Second, we evaluated McFarlane's method of the 1950s, which uses iodine monochloride (ICl) at pH 8.5. Both produce mono (3‐iodo‐tyrosine) and di‐iodo tyrosine (3,5‐iodo‐tyrosine), in varying ratios depending on reaction time and iodine concentration, with the higher pH of McFarlane's reaction also contributing toward histidine iodination.^[^ ^35^ ^]^ Tyrosine was deemed a suitable target for iodination due to its low but consistent molar fraction (0.3 and 2%) of the amino acid residues of collagen across the tissues of various mammalian and aquatic species.^[^ ^36^, ^37^ ^]^ Unlike proline and glycine which are arranged periodically along the whole length of collagen, tyrosine is concentrated at the end, and not known to be involved in the hydrogen bonding interactions that determine collagen's triple‐helical structure, making it a suitable labeling target with low potential for disrupting stability and function.^[^ ^38^ ^]^ Due to this typically low abundance in protein, much recent work has gone into developing tyrosine‐targeted click chemistry approaches for selective functionalization of small soluble proteins, though this has not yet extended to the modification of large protein scaffolds.^[^ ^39^, ^40^, ^41^, ^42^, ^43^, ^44^ ^]^ To demonstrate the retention of relevant material and biological properties post‐labeling we present the results of mechanical testing, scanning electron microscopy (SEM), Energy Dispersive X‐ray spectroscopy (EDS), cell growth assays and ex vivo histological analysis.

To illustrate the effectiveness of our labeling method we enhanced the radiological visibility of hernia meshes, which is currently poor and known to affect their clinical management.^[^ ^45^, ^46^ ^]^ Mesh‐based hernia repair is the most‐performed surgical technique worldwide, at ≈20 million annual operations globally.^[^ ^47^ ^]^ Though the average complication rate of this procedure is high at ≈10%, common comorbidities such as chronic heart disease, diabetes, obesity, and infection, can further increase relapse or complication rates above 60% in affected populations.^[^ ^48^, ^49^ ^]^ In such cases unwanted mesh behavior can include perforation, tearing, detachment, infection, and migration, typically requiring either removal, repair, or reattachment. Yet the similarity of biological meshes to native tissue currently hinders detection during surgery or with CT.^[^ ^45^ ^]^ The ability to non‐invasively and longitudinally visualize mesh placement and morphology in patients over time should therefore make a useful contribution to the diagnosis of complications, and surgical planning to correct these. To this end, we track an iodinated collagen‐based hernia mesh in vivo up to three months post‐implantation, with a comparable biological response of the labeled mesh measured versus an unmodified off‐the‐shelf control.

In summary, this method is a fast, simple, and cost‐effective route to label pre‐formed protein‐based biomaterials for detection with the most widely used clinical imaging modality, X‐ray CT. We show that this labeling method is achievable under mild and scalable conditions, maintaining the biocompatibility and strength of the starting material. To demonstrate utility, we label a clinically approved, commercially available collagen‐based hernia mesh, and quantify improved contrast in a mouse model up to three months post‐implantation using non‐invasive X‐ray CT imaging. Broadening the potential applications, we also demonstrate that this labeling method enables X‐ray CT detection of a range of other protein‐based materials used surgically and in tissue engineering, including silk sutures and egg shell membranes.

## 2 Results

### 2.1 Enhanced Visibility of Protein Scaffolds

**Figure 1:**
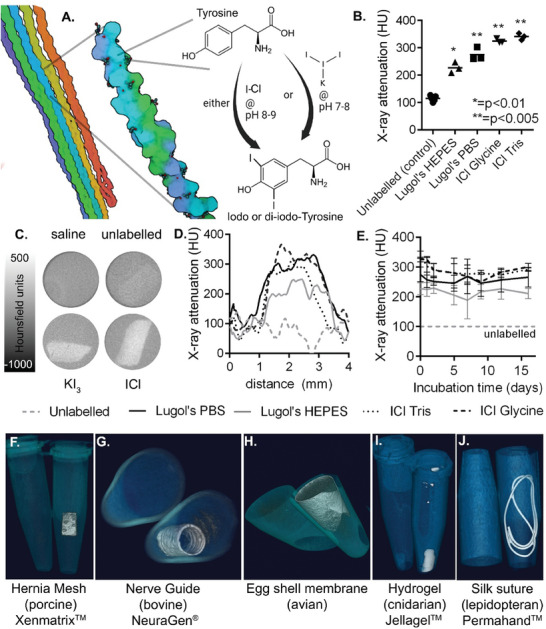
A) Schematic of collagen labeling showing color‐coded collagen fibrils and chains respectively, with iodination of a tyrosine residue following reaction with KI_3_ or ICl. B) Increased radiopacity of porcine collagen scaffolds (Xenmatrix) versus unlabeled controls at 50 kVp, following Potassium Tri‐iodide (Lugol's) reaction or Iodine monochloride reaction (ICl) in the indicated buffers (unpaired, two‐tail *t*‐test). C) Representative X‐ray CT sections showing saline only, unlabeled and labeled (KI_3_ in PBS, and ICl in Glycine) hernia mesh samples. D) Cross‐sample intensity profile shows relative homogeneity of sampling for each of the reaction conditions. E) Increased radiopacity was retained up to 16 days of incubation at 37 °C in human serum. F–J) 3D rendered X‐ray CT images of unlabeled (left tube) and labeled (right tube, potassium tri‐iodide reaction in PBS) Hernia mesh, nerve guide, egg‐shell matrix, jellyfish collagen hydrogel, and silk suture.

The efficacy of both candidate iodination reactions (Potassium Triiodide–KI_3_, and Iodine Monochloride–ICl) was evaluated on samples of a representative clinically approved collagen‐based scaffold produced from decellularized porcine dermis (XenMatrix hernia mesh, Bard). Two buffers were compared per reaction, chosen for their suitable pH range and established use in protein biochemistry. Phosphate buffered saline (PBS) and HEPES for the KI_3_ method (at pH 7.4), and Glycine and Tris buffers for the ICl method (at pH 8.5),^[^ ^50^ ^]^ see **Figure** **1**A. Each reaction/buffer combination increased the radiopacity of labeled materials following 24‐h incubation and removal of unbound iodine, with significantly increased Hounsfield Units (HU) measured with X‐ray CT versus unlabeled control samples (Figure 1B,C). For the KI_3_ method, PBS showed superior labeling to HEPES buffer, consistent with its previously reported acceleration of tyrosine iodination.^[^ ^51^ ^]^

Assuming an iodine radiopacity of 14–16 HU per mg Iodine/mL,^[^ ^52^ ^]^ a tyrosine weight fraction of 1.96% for porcine collagen type 1 (UniProt ref: A0A287BLD2), and a protein weight/volume fraction for decellularized porcine dermis of 40%,^[^ ^53^ ^]^ then total di‐iodination of tyrosine residues would be expected to increase radiopacity by 154–175 HU. This was comparable to the increase in HU measured for KI_3_ with HEPES buffer (112 HU) and PBS (160 HU), see Figure 1B. Fluorescence spectroscopy gave a characteristic 300 nm peak for tyrosine in control (unmodified) samples, which decreased significantly in intensity by 98% following labeling with either KI_3_/PBS or ICl/Glycine reactions (Figure S1 (Supporting Information), *n* = 3, *p* < 0.005 two‐tailed *t*‐test). This is consistent with previously reported decreases at the 300 nm tyrosine fluorescence peak following iodination of tyrosine residues,^[^ ^54^, ^55^ ^]^ and complete conversion of tyrosine to di‐iodotyrosine in soluble collagen under similar conditions (56).

Interestingly the iodine monochloride reaction showed significantly higher levels of CT contrast (*p* < 0.05 vs KI_3_/PBS, students two‐tailed *t*‐test; 210 HU for Glycine buffer, 226 HU for Tris buffer), suggesting additional modification sites. This is consistent with previous reports of histidine iodination occurring in addition to tyrosine iodination above pH 8,^[^ ^35^, ^57^ ^]^ with mono‐iodination of histidine in addition to di‐iodination of tyrosine giving a predicted increase of 220–251 HU.

Homogeneity of labeling, and iodine incorporation throughout the scaffold, were investigated by plotting line profiles of signal intensity for samples labeled with each of the four reaction combinations (Figure 1D). This showed elevated X‐ray attenuation across the labeled scaffolds, with some variation in intensity consistent with their porosity. To assess labeling stability, samples were incubated at 37 °C in human serum for 16 days, with radiopacity measured at intervals using X‐ray CT (Figure 1E). No contrast reduction was identified over this period, suggesting stable iodine retention within the material under biologically relevant conditions.

To ensure greater selectivity of modification and to minimize alterations to biological function, the milder conditions of the potassium tri‐iodide method were chosen for use throughout the rest of this study, unless otherwise indicated. Potassium triiodide, in the form of Lugol's Iodine as used here, also has the advantage of being clinically established as an antiseptic agent, further increasing the feasibility of translation. Investigation of the kinetics of iodination with KI_3_/PBS showed that 80% of the peak labeling was achieved in 45 min (Figure S2, Supporting Information), reaching saturation after 3 h. Contrast from the labeled collagen scaffold was found to be retained in saline at room temperature over a period of 17 months (Figures S3 and VideoS1, Supporting Information).

To demonstrate versatility, we next labeled four additional protein‐based materials of interest (Figure 1F–J). NeuraGen (Figure 1G; Video S2, Supporting Information) is an FDA‐approved hollow tube formed of porous bovine collagen, implanted to repair peripheral nerve damage.^[^ ^58^ ^]^ However, surgical implantation is challenging, and implants must be located and removed when unsuccessful.^[^ ^59^ ^]^ Labeling with KI_3_/PBS increased radiopacity by ≈200 HU (Figure 1G), and labeling stability was confirmed up to 15 months at room temperature in saline (Figure S4, Supporting Information). We also labeled egg‐shell membrane (ESM) (Figure 1H), which contains a number of proteins alongside type I and type IV collagen.^[^ ^60^ ^]^ Again, an increase of ≈200 HU was achieved with KI_3_/PBS (Figure 1H), and labeling stability was preserved over 15 months (Figures S5.and VideoS3, Supporting Information). We next demonstrated that a commercially available hydrogel of “Type 0” jellyfish‐derived collagen could also be labeled and imaged with an increased radiopacity of ≈280 HU (Figure 1I). This material has been suggested as more sustainable alternative to the established porcine and bovine collagen sources.^[^ ^61^, ^62^ ^]^ Finally, we labeled a commercially‐available silk‐suture (Ethicon, 3‐0), which showed an increase in HU of over ≈1400 (Figure 1J; Figure S6 and Video S4, Supporting Information), consistent with the unusually high tyrosine content of silkworm (Bombyx mori) silk, at five to sixfold that of collagen (UniProt: P05790 · FIBH_BOMMO).

### 2.2 Surface Morphology, Biocompatibility, and Material Strength Are Preserved Post‐Iodination

**Figure 2:**
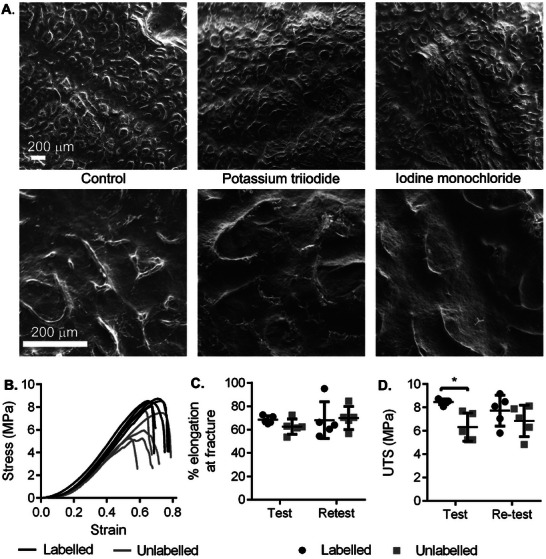
A) Surface morphology is comparable between control (unlabeled) collagen scaffolds and those labeled using Potassium triiodide and Iodine monochloride methods, as seen with scanning electron micrographs at two levels of magnification. B) Stress–strain curves of labeled and unlabeled meshes, one line per replicate. C) No significant difference was found between labeled and unlabeled scaffolds in elongation percentage at the point of fracture (two‐tailed *t*‐test). D) Labeled meshes required a significantly higher max force (ultimate tensile strength, UTS) to induce fracture in primary tests, but not retests (*p* < 0.05, two‐tailed *t*‐test), indicating increased strength. *N* = 5, lines show the mean, error bars show the standard deviation.

To demonstrate the preservation of native surface structure, collagen scaffolds (Xenmatrix hernia mesh) labeled with potassium tri‐iodide (PBS buffer), and iodine monochloride (glycine buffer) were analyzed using SEM and compared to unmodified samples. No change in large‐ or small‐scale structure was identified after labeling with either reaction (**Figure** **2**A), consistent with the mild reaction conditions. Energy‐dispersive X‐ray spectroscopy (EDS) analysis confirmed iodine was only present in labeled samples (Figures S7–S10, Supporting Information).

To investigate scaffold biocompatibility, mouse MSCs were seeded onto labeled (KI_3_/PBS) and unlabeled collagen scaffolds, and their metabolism was recorded as light output by the ATP‐dependent luciferase reaction up to 14 days. Cell doubling times during the exponential growth phase showed no significant difference between the labeled (22.93 h ± 1.03 SD) and unlabeled (24.38 h ± 3.23 SD) materials respectively (Figure S11, Supporting Information; *p* = 0.49, two‐tailed *t*‐test). Similarly, no significant difference in light output was found between conditions at any stage up to and including the final timepoint (Figure S11, Supporting Information), indicating that labeling had no effect on the size of the supportable cell population. To confirm this and investigate cell distribution on the materials, samples were sectioned at 14 days post‐seeding and stained with the nuclear dye DAPI. Comparable surface localized monolayer formation was seen on both labeled and unlabeled scaffolds (Figure S12, Supporting Information). As the use of high concentrations of intravenous iodinated contrast agents has previously been linked to increased risk of kidney disease,^[^ ^63^ ^]^ we also evaluated the growth rate of a human kidney‐derived cell line (HEK293T) on the iodinated scaffolds as per above. Again, no significant difference was found in cell growth rate between conditions (Figure S13, Supporting Information), consistent with the stable retention of iodine on the scaffolds (Figure 1E; Figure S3,Supporting Information).

Dynamic mechanical analysis testing of control and KI_3_ (PBS) labeled collagen scaffolds (Xenmatrix hernia mesh) showed comparable stress–strain curves (Figure 2B), and percent elongation at fracture (Figure 2C). Ultimate tensile strength (UTS) was however increased significantly in labeled meshes (Figure 2D; 8.46 ± 0.24 (SD) MPa vs 6.32 ± 1.19 MPa, *p* < 0.005 two‐tailed *t*‐test), though remained within the range of 2.53 ± 0.25 MPa to 28.54 ± 1.99 MPa reported for commercially available bovine and porcine‐derived biological hernia meshes.^[^ ^64^ ^]^ This difference was not significant upon re‐testing the material (7.72 ± 1.31 MPa vs 6.84 ± 1.33 MPa, *p* = 0.32). Similarly, a small but significant increase in Young's modulus was measured upon labeling from 15.3 to 18.1 MPa (*p* < 0.01, student's two‐tailed *t*‐test), which again was found to be non‐significant during the re‐test condition. FT‐IR and Differential Scanning Calorimetry showed comparable spectra for labeled and unlabeled materials, albeit with minor peak shifts presumably due to the incorporation of iodine (Figures S14 and S15, Supporting Information).

As silk is typically one to two orders of magnitude stronger than collagen, and with much higher tyrosine content, we also investigated the preservation of its material properties post‐iodination. Consistent with the collagen samples, surgical silk sutures (as shown in Figure 1J) showed no morphological changes via SEM (Figure S12, Supporting Information) when labeled with either the KI_3_ or ICl methods. Mechanical properties were also preserved, with both the UTS and Young's modulus of labeled samples showing no significant difference when compared to unlabeled controls (Figures S17 and S18, Table S1, Supporting Information).

### 2.3 Iodinated Hernia Meshes Remain Trackable up to 3 Months In Vivo

**Figure 3:**
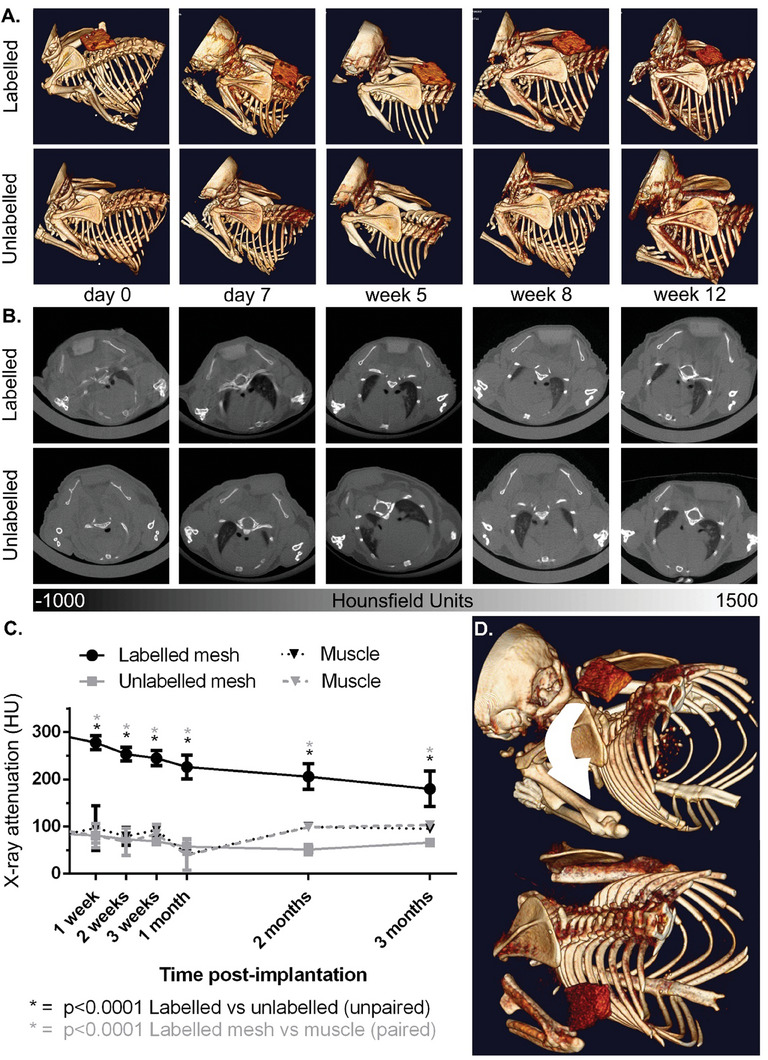
Iodinated hernia mesh samples (Xenmatrix) show increased visibility on X‐ray CT versus unlabeled samples and muscle over 3 months post‐implantation on A. 3D reconstructions, B. axial CT cross sections, and C. region of interest quantification of X‐ray attenuation (HU). Holm‐Šídák multiple comparison tests show significantly higher radioapacity of labeled meshes versus muscle (paired), and versus unlabeled samples (unpaired) at all time points. *N* ≥ 3 per time point, error bars show standard deviation. D. CT renderings showed lateral mesh migration in one animal post implantation.

To demonstrate clinical utility, we implanted immunocompetent mice subcutaneously with labeled (KI_3_/PBS) and unlabeled samples of the collagen‐based hernia mesh (Xenmatrix). Subsequent X‐ray CT imaging extending to 3 months (12 weeks) post‐implantation showed significant differences in radiopacity (HU) between labeled and unlabeled meshes (*p* < 0.0001), and between labeled meshes and muscle within the same animal (*p* < 0.0001), confirming radiopacity was sustained over time (**Figure** **3**A–C; Videos S5 and S6, Supporting Information). No difference in radiopacity between unlabeled meshes and muscle was found (*p* > 0.05), illustrating the existing challenge when attempting to discriminate commercially available meshes from surrounding tissue. Highlighting utility to detect material migration from implantation site, one labeled mesh had moved laterally between implantation and subsequent imaging 1‐week post‐implantation (Figure 3D), a phenomenon that can occur clinically when the mesh detaches from surrounding tissues.

### 2.4 Preserved Visibility Using Clinically‐Relevant X‐Ray Doses

**Figure 4:**
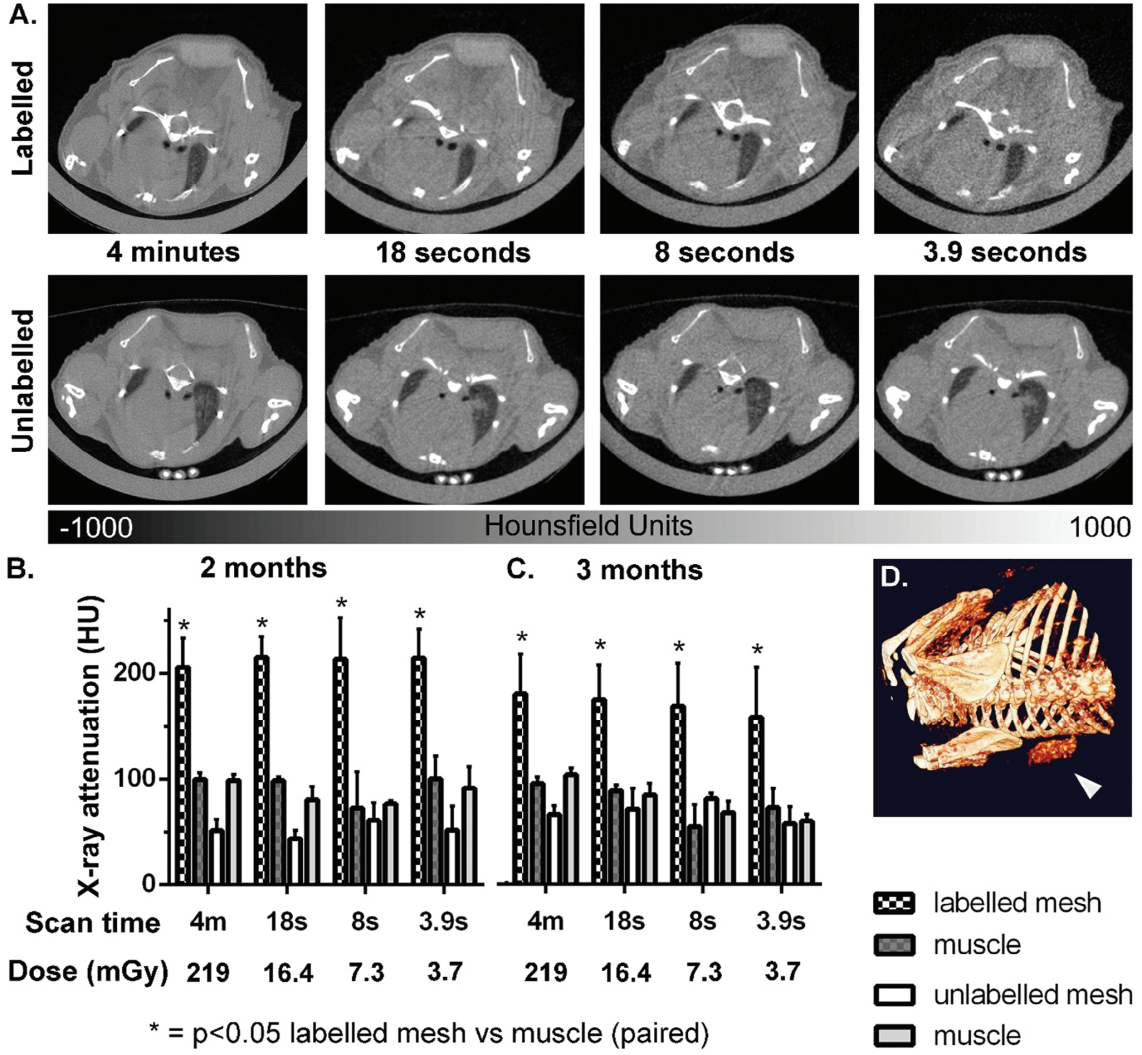
Labeled meshes retained their increased visibility versus muscle and control mesh with decreasing scan times, as shown here with A) axial CT sections at 3 months post‐implantation, and region of interest quantification of radiopacity at B) 2 months, and C) 3 months post‐implantation, with Holm‐Šídák multiple comparison tests. *N* ≥ 3 per time point, error bars show SD. D) Laterally migrated mesh visualized on a 3D rendered 18 s CT scan at 3 months post‐implantation using a clinically relevant X‐ray dose of 16.4 mGy.

To assess the feasibility of imaging the labeled material using clinically relevant X‐ray doses, we imaged implanted meshes with a range of scan times at 2 and 3 months post‐implantation. At all scan times between 4 min and 4 s, labeled meshes showed good visibility on CT sections (**Figure** **4**A) and a significantly higher HU than both muscle and unlabeled meshes following region of interest analysis (Figure 4B). To put these results into perspective, average clinical volumetric CT doses of 25 and 17 mGy (with 1 mGy equalling 1 Joule of energy absorbed per kg) were reported for abdominal imaging in Europe and the US respectively,^[^ ^65^ ^]^ which corresponded most closely to our 18 s/16.4 mGy scan. This X‐ray dose was sufficient to produce comparable quantification of intensity (HU) to the 4‐min scan for mesh and muscle, and to visualize the migrated mesh at 3 months post‐implantation in 3D following intensity‐based 3D rendering (Figure 4D).

### 2.5 Biocompatibility of Visible Mesh

**Figure 5:**
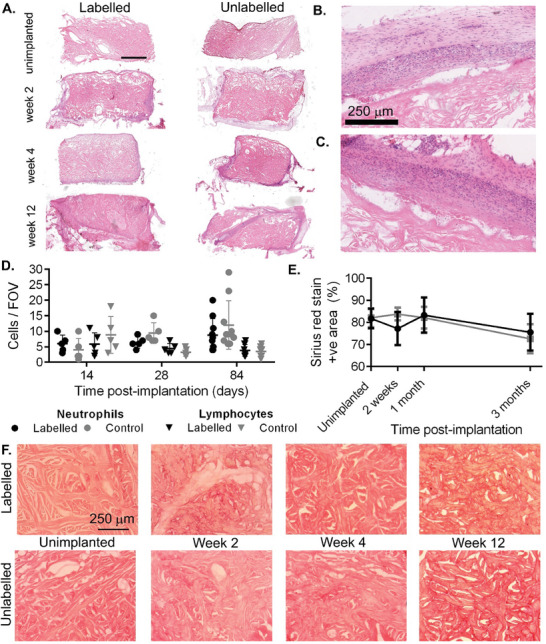
A) Macroscopic H and E stained sections of explanted meshes at 2, 4, and 12 weeks post‐implantation. Scale bar = 1 mm. Representative sections showing comparable infiltration of endogenous tissue at the mesh boundary at 12 weeks for B) labeled, and C) unlabeled meshes. D) No significant difference was found between mean neutrophils and lymphocyte counts at 40× magnification in H and E stained tissue sections at 2, 4, and 12 weeks post‐implantation (two‐tailed unpaired *t*‐test, error bars show SD). Labeled meshes showed comparable collagen coverage to unlabeled meshes up to 3 months post‐implantation, as seen with E) Threshold quantification and F) representative images of the collagen‐stained area (*p* > 0.05 for all time points, two‐tailed unpaired *t*‐test, *n* ≥ 5 regions of interest) and F. representative microscopic images showing Sirius‐red stained sections.

To assess the effect of iodination on the immune response to implanted meshes, samples were explanted at 2, 4, and 12 weeks, and H and E staining was performed on histological sections (**Figure** **5**A). Neutrophil and lymphocyte numbers were counted at 40× magnification by a trained histopathologist on *n* ≥ 5 sections per time‐point and sample (Figure 5B), showing comparable numbers between labeled and unlabeled meshes at each time point (*p* > 0.05 two‐tailed unpaired *t*‐tests). Infiltration of native tissue was comparable in labeled and unlabeled meshes, with representative sections at 12 weeks post‐implantation shown in Figure 5C,D.

### 2.6 Preserved Mesh Integrity

To investigate the biological response to labeled meshes further, samples were explanted at 2, 4, and 12 weeks, and stained using Picro Sirius Red stain for collagen (Figure 5E,F). Labeled and unlabeled meshes appeared similar at each time point, with quantification of stained area not significantly different in coverage between mesh types at any time point. No change in overall mesh volume for labeled meshes was measured based on semi‐automatic intensity‐based segmentation of the labeled implants (Figure S19, Supporting Information), consistent with the overall preservation of collagen coverage seen histologically.

## 3 Discussion

This study introduces an innovative approach to labeling protein‐based biomaterials for in vivo X‐ray CT imaging, enabling long‐term tracking of these materials with minimal alteration of their native properties. To do this we have exploited an obsolete radiochemistry method, adapting it for the first time to produce radiopaque biomaterials by combining stable iodine isotopes and macroscopic protein scaffolds. This builds on the growing interest in labeling biological and biodegradable polymers for non‐invasive imaging,^[^ ^25^ ^]^ including incorporation of radionuclides for nuclear imaging,^[^ ^66^, ^67^, ^68^ ^]^ contrast agents for MRI,^[^ ^69^, ^70^, ^71^, ^72^, ^73^ ^]^ as well as high‐Z elements for CT imaging.^[^ ^27^, ^71^, ^74^, ^75^, ^76^, ^77^ ^]^ Compared to radionuclide‐based polymer labeling methods,^[^ ^56^, ^58^ ^]^ CT imaging offers greater resolution, while overcoming the limitations associated with label half‐life, thus enabling imaging at significantly later time points post‐transplantation. This feature is particularly valuable when considering implants like hernia meshes, which need to remain in place for extended periods. Compared to nanoparticle‐based labeling approaches for CT and MRI, our method also avoids the need to incorporate contrast agents during material synthesis,^[^ ^27^, ^73^, ^76^, ^77^ ^]^ thereby opening up the possibility of labeling scaffolds based on decellularized tissue.

Our in vivo results showed clear visibility of labeled hernia meshes above both endogenous muscle and control unlabeled mesh samples. This was maintained over a biologically relevant time frame of three months, and under X‐ray doses at and below those typically measured in the clinic. With hernia meshes being among the most established clinically used biomaterials (≈20 million implantations per year globally ^[^ ^47^ ^]^), their improved radiological visibility demonstrated here could potentially improve healthcare outcomes for a considerable population. Complication rates for implanted meshes often exceed 10%, reaching above 60% where comorbidities such as diabetes, obesity, chronic heart disease, and infection, increase relapse or complication risks.^[^ ^48^, ^49^ ^]^ This can include infection, perforation, tearing, detachment, and mesh migration, typically necessitating either removal, reattachment, or repair. The similarity of biological meshes to native tissue frustrates their discrimination when using CT, or indeed visually during surgery.^[^ ^45^ ^]^ The ability to non‐invasively and longitudinally visualize mesh placement in patients over time should therefore make a useful contribution to diagnosis of complications, and surgical planning to correct these.

Currently, the only commercially available contrast‐enhanced hernia mesh is a synthetic (PVDF–polyvinylidene difluoride) material, loaded with iron‐oxide nanoparticles that reduce signal intensity on MR imaging.^[^ ^78^ ^]^ While this labeling and imaging approach increases the visibility of the implant and its shrinkage,^[^ ^79^ ^]^ MRI is used much less frequently than CT in the clinic for hernia patients.^[^ ^45^ ^]^ In particular, smaller bores and lower patient weight limits versus CT mean that obese patients, a significant target for mesh therapy, often cannot be imaged with MRI.^[^ ^80^, ^81^ ^]^ In terms of spatial resolution, CT imaging is comparable to MRI in the clinic, as well as typically being cheaper, faster, and more widely available.^[^ ^82^ ^]^ As this bulk‐iodination technique is not compatible with meshes made from synthetic polymers, their labeling for CT remains a source for future research. However, the increased foreign‐body response, and resultant fibrosis and chronic pain associated with synthetic meshes highlights the benefits of protein‐based alternatives that better match the properties of endogenous tissue.^[^ ^1^, ^5^, ^83^ ^]^ The biological and mechanical features of the hernia however are complex, and not fully recreated in the animal model used here—lacking as it did the disease‐associated increases in mechanical strain, inflammation, and native tissue remodelling processes—all of which vary clinically between sites and size of hernia occurrence. Though we used immunocompetent mice and confirmed retention of mechanical properties, future safety studies should be performed in more clinically relevant environments.

By using a fast amino‐acid selective reaction, our approach shares many of the benefits of recent click chemistry strategies for protein labeling, which have been used to incorporate dyes, nanoparticles, and metal chelates for imaging.^[^ ^84^, ^85^, ^86^ ^]^ However, these reports have typically been restricted to functionalising small soluble proteins, with little having been done previously on large pre‐formed protein scaffolds. Here there has also been a trend toward tyrosine‐specific strategies.^[^ ^39^, ^40^, ^41^, ^42^, ^43^, ^44^ ^]^ Being less abundant than the more established targets of cysteine and lysine, tyrosine provides a route toward more selectively‐modified products, with less potential to disrupt biological function.^[^ ^41^ ^]^ In this study however, we have modified tyrosine directly with the imaging agent, and as such circumvented the need for a bulkier, higher molecular weight carrier molecule for iodine as is necessary when using a functional linker to attain specificity.^[^ ^87^ ^]^ Working only with iodine and not a range of imaging agents, however, our labeling approach does lack the versatility of such click‐chemistry strategies, or indeed of newer chelate‐free labeling methods.^[^ ^88^, ^89^ ^]^ Yet it also avoids the limitations of poor commercial availability and complex synthesis associated with many bioconjugates or click chemistry reagents, increasing usability. Though we evaluated two of the simplest methods of tyrosine iodination, an ethanol‐based approach has previously shown efficient iodination of complex biological polymers such as wool, albeit with longer reaction times.^[^ ^90^ ^]^ Future comparison with other reaction conditions might reveal more efficient alternatives.

X‐ray visible collagen scaffolds have also recently been made by tethering gold nanoparticles to peptides using the EDC/NHS coupling reaction,^[^ ^27^ ^]^ in a similar manner to a previous study incorporating iron oxide nanoparticles for MRI.^[^ ^73^ ^]^ This provided information on scaffold degradation using ex vivo CT. However, lacking the specificity of some of the aforementioned click‐chemistry approaches, EDC/NHS coupling can target the protein c‐terminus, aspartate, and glutamate, which due to their greater abundance in collagen could result in a ≈20‐fold higher degree of modification than a tyrosine‐specific reaction, risking greater alteration of biological function. Moreover, the cost of gold nanoparticles needed for larger scale visualization would likely preclude whole‐mesh labeling for clinical application,^[^ ^91^ ^]^ limiting their use as markers in specific spatially‐organized sub‐volumes of the material.^[^ ^27^ ^]^ In contrast, the method employed in our study, provides a more specific and scalable approach to achieving X‐ray visibility, with the incorporation of fewer and lower MW modifications minimising material alteration compared to nano or micro‐particle‐based methods previously described for MR,^[^ ^70^, ^71^, ^73^ ^]^ and CT.^[^ ^27^, ^71^, ^92^ ^]^

We also showed that this labeling method was compatible with other protein biomaterials including silk and eggshell membrane (ESM), providing ample scope for future implementation outside the field of hernia repair. ESM is typically discarded as a waste product by the food industry, but due to its favorable biocompatibility and material properties has been suggested as a sustainable alternative to bovine and porcine sources of collagen. Various recent studies illustrate the versality of ESM, showing repair of damaged ear drum, nerves, cartilage, and cardiovascular defects.^[^ ^93^, ^94^, ^95^ ^]^ Though silk is most commonly used as a surgical material, new material processing techniques are emerging,^[^ ^96^ ^]^ together with a variety of cellular and acellular regenerative therapies including tendon and ligament grafts, cardiac patches, and vascular constructs.^[^ ^97^ ^]^ Our proof‐of‐concept labeling demonstrated that silk exhibits a sixfold higher radiopacity than decellularized porcine dermis, in line with the sixfold higher tyrosine content of silk versus collagen. While previous attempts to produce radiopaque silk have decreased its tensile strength by several fold,^[^ ^26^ ^]^ our method was found to retain its prized mechanical properties. With comparable or higher tyrosine content in other fibrillar proteins of interest in biomaterials, such as elastin (≈2%, UniProt: P15502) and keratin (2.7%, UniProt: P02533), similar results might be expected to those achieved here with collagen.

Though we have focused here on the potassium tri‐iodide reaction, this might be unfavorable for proteins with low tyrosine content, or where its modification disrupts function. Here, histidine labeling with iodine monochloride would warrant further investigation, or indeed the adaptation of more recent radio‐iodination chemistries using the lysine and N‐terminus specific Bolton‐Hunter reagent.^[^ ^30^, ^98^ ^]^ A further advantage of such an approach would be the greater in vivo stability of its newer analogues,^[^ ^99^ ^]^ designed to resist the action of endogenous de‐iodinases. Yet further improvements to contrast could be made by attaching branched or poly‐iodinated agents^[^ ^100^ ^]^ to tyrosine or other selected amino acids via clickable linkers.^[^ ^39^, ^40^, ^41^, ^42^, ^43^, ^44^, ^84^, ^85^, ^86^ ^]^ This latter approach could also compensate for the relatively modest signal enhancement achieved here, in comparison to the orders of magnitude higher percentage weight of iodine previously incorporated into synthetic polymers such as methacrylates,^[^ ^101^ ^]^ polyurethanes,^[^ ^102^ ^]^ and polyesters.^[^ ^103^ ^]^

In conclusion, we present a fast, cost‐effective, and scalable method to produce X‐ray visible protein scaffolds, relying on bulk iodination of tyrosine residues. The utility of this approach was demonstrated on a commercially available hernia mesh comprized of decellularized porcine dermis, which showed higher radiopacity up to three months post‐implantation, compared to unlabeled stock materials and native tissue. Both labeled and unlabeled materials showed otherwise similar behavior in vivo, indicating the retention of biocompatibility post‐labeling. Labeling was also demonstrated on a range of other commercially‐available biomaterials, illustrating its potential for tracking protein‐based tissue engineering constructs in general. Implanted meshes remained visible in vivo at scan times and radiation doses equivalent to and below those used clinically, further demonstrating the translatability of this technique.

## 4 Experimental Section

### Material Labeling

For the triiodide labeling method, 5 × 10 mm hernia mesh samples (Xenmatrix, Bard), or 40 × 40 mm Egg shell membrane were added to either 1 mL of HEPES (200 mm, pH 7.4) buffer (4‐(2‐hydroxyethyl) −1‐piperazineethanesulfonic acid, Sigma Aldrich), or PBS (phosphate buffered saline solution, pH 7.4, Gibco) with 400 µL Lugol's iodine solution (SLS, CHE2380, 38 mm), and incubated for 24 h. All samples were washed with 3 × 10 mL changes of fresh saline (0.9% w/v NaCl solution) with 1 h incubation each on a tube rotator (20 rpm), with two final 24 h incubations also with rotation. For the iodine monochloride (McFarlane's) labeling method, mesh samples (5 × 10 mm) were added to either 1 mL of 200 mm pH 8.6 glycine buffer (Reagent Plus, ≥99% Sigma–Aldrich) or 200 mm pH 8.6 Tris‐HCl buffer (Trizma, Sigma–Aldrich), containing 10 uL of iodine monochloride (Alfa Aesar, 39104), and incubated for 24 h, before washing as above. Neuragen nerve guide (Integra Life Sciences) was labeled as above with Lugol's/PBS in 1.5 cm lengths, before washing as above. Silk sutures (Permahand, Ethicon, 3‐0) were de‐waxed for 5 min in xylenes, washed twice with 70% ethanol, and twice with PBS, and labeled with the above Lugol's/PBS or ICl Glycine method, before washing as above. Purified collagen (Research Grade Jellyfish Collagen, Jellagen), 100 µL at 4 mg mL^−1^, was labeled as above with the Lugol's/PBS method, and precipitated with ethanol, before washing as above.

### Egg Shell Membrane Extraction

Egg shell membranes (ESM) were extracted by a method adapted from Mensah et al.^[^ ^104^ ^]^ Briefly, after washing with deionized water (DI), fresh chicken eggs were immersed in 0.5 m acetic acid for 44 h at room temperature to dissolve the calcium carbonate (CaCO_3_) shell. After CaCO_3_ shell dissolution was complete, the eggs were washed again using DI and the membrane was perforated to empty the albumen and yolk. A final washing step was done before storage in PBS to prevent dehydration.

### Labeling Stability

To determine labeling stability in human serum, samples produced using each of the four labeling methods described above (Lugol's/HEPES, Lugol's/PBS, ICl/glycine ICl/Tris) were incubated in 1.5 mL of human serum (Standard Pooled Human Serum, Cambridge Bioscience) at 37 °C for 16 days. Samples were imaged at 0, 1, 2, 5, 7, 9, 12, and 16 days post labeling. For long‐term stability, samples (Neuragen, ESM, and Xenmatrix hernia mesh) were incubated at room temperature in saline (0.9% w/v NaCl), and imaged up to 17 months post‐labeling. Retention of contrast was assessed using a PET‐CT system (Mediso nanoScan) with a semi‐circular scan at 50 kVp with 170 ms exposure, 720 projections, 1:4 binning, and medium FOV setting. Region of interest analysis was performed using VivoQuant software (Invicro) to quantify X‐ray attenuation.

### SEM and EDS Spectroscopy

Collagen scaffold samples were mounted on specimen stubs fitted with adhesive carbon pads, sputter‐coated with carbon and examined using a Zeiss Evo50 (Oxford Instruments, Cambridge, UK) scanning electron microscope, with micrographs obtained at an acceleration voltage of 20 kV. Point EDS spectra were acquired using and Oxford Instruments x‐act EDS detector running INCA software. Silk samples were fixed to adhesive 12 mm carbon tabs (Agar Scientific, Stansted, UK) pre‐mounted onto 0.5 aluminium spectrum stubs (Agar Scientific, UK), and imaged using a field emission scanning electron microscope, (FE)SEM, (Zeiss, EVO HD, Jena, Germany) with operation voltage of 5 kV. Samples on the stubs were sputter‐coated with 95% gold and 5% palladium (Polaron E500, Quorum Technology, UK) and imaged at magnifications of x500 and X1K.

### Cell Growth Assays

Hernia mesh samples (Xenmatrix) were labeled as above using KI_3_/PBS and cut to 7 mm diameter, sterilized in 70% ethanol, and washed twice in sterile PBS (Gibco) before insertion at one per well into opaque white 96 well cell culture plates (Pierce, Thermo Scientific). Luciferase‐expressing D1 ORL UVA (ATCC CRL‐12424) mouse mesenchymal stem cells (a kind gift from Dr Arthur Taylor, University of Liverpool), or luciferase‐expressing human embryonic kidney cells (HEK293T), were seeded at 2000 per well, and grown at 37 °C in 200 µL DMEM (Gibco) with 10% foetal bovine serum (FBS, Gibco), with Penicillin–Streptomycin (Gibco). Light output was recorded using a bioluminescence imaging device (IVIS Spectrum, Perkin Elmer) over 10 s using small binning at 20 min post luciferin addition (10 µL per well, 15 mg mL^−1^). Data was analysed using Living Image software (Perkin Elmer) with circular regions of interest placed on each well, and the average photons per second recorded for each well. A fresh media change was given following each bioluminescence assay. Following the 14‐day timepoint, meshes grown with MSCs were removed and embedded in optimal cutting temperature medium (OCT, Thermo Fisher), before cryosectioning at 10 µm onto glass slides (Superfrost Plus, Thermo Scientific). Equivalent samples were also prepared from labeled and unlabeled meshes that had not been seeded with cells. Nuclei staining was done using DAPI‐containing mounting media (Fluoroshield, Abcam), and imaged using a fluorescence microscope (Zeiss Axiovert 5) with a 20× objective and brightfield and DAPI settings.

### Tensile Strength Testing

Tensile strength of labeled and unlabeled hernia mesh samples (*n* = 5) was evaluated at room temperature using a uniaxial testing device (AGS‐X 200 kN, Shimadzu), with the following measurement of dimensions prior to testing: 8.298 ± 0.50 mm wide, 14.9 ± 0.24 mm long and 2.16 ± 0.08 mm thick. Each specimen was clamped an average of 3.12 ± 0.44 mm on each end of the long axis before starting the test. During the test the samples were pulled apart at 5 mm min^−1^ in the Y‐axis while recording force and displacement until failure, which was defined as a drop of 50% in the force needed to continue pulling the sample apart. After the initial “fresh” test, the samples were trimmed at the fracture point and rehydrated in PBS for 20 min prior to clamping and retesting under the same conditions (labeled “Re‐test” in the results). Silk sutures (80 mm long and 0.2 mm diameter) were double knotted at each of a metal 75 mm mending plate and the space from knot to knot was measured as the gap Gauge length (19.46 ± 3.16 mm). Both mending plates were clamped on each end of the long axis and the gap increased enough to slightly tense the suture with minimal force before starting the tensile test. During the test, the sutures were pulled apart at a rate of 5 mm min^−1^ in the Y‐axis while recording force and displacement until failure, which was defined as a 50% drop in the force.

### Animals

Female C57BL/6 mice were purchased from Charles River were 3 weeks old at the point of implantation. Mice were housed five per individually ventilated cage, at 21 °C with normal day/night cycles. All animal studies were licensed under UK Home Office regulations and approved by the UCL Biological Services Ethical Review Committee, with all regulations were in compliance with UCL experimentation guidelines and regulations.

### Mesh Implantation

Mice were anesthetized in a prone position with 2% isoflurane in 100% O_2_, and body temperature was maintained at 37 °C via a heating pad. A 1 cm longitudinal incision was made using surgical scissors, and sterilized 4 × 4 mm squares of Xenmatrix mesh (BD Bard) were inserted subcutaneously under the scruff (*n* = 10 each for labeled and unlabeled mesh). The skin was closed using Mersilk 3/0 suture (ETHICON, W502H). Mice were kept in a recovery chamber at 37 °C after surgery for 20 min.

### In Vivo Imaging

Mice were anesthetized with 2% isoflurane in 100% O_2_, and maintained under anaesthesia during imaging with a preclinical micro‐CT device (Quantum GX2, PerkinElmer). Mice were scanned at 0, 7, 14, 21, 28, 35, 56, and 84‐day timepoints with 4‐min high‐resolution scans (Voxel size 72 µm, 90 kVp, filters Cu 0.06 + Al 0.5). On the days 56 and 84 timepoints mice were also imaged with 18‐s (Standard), 8‐s (High speed), and 3.9‐s (High speed) scans for comparison, also at 90 kVp with Cu 0.06 + Al 0.5 filters. 3D ROI analysis was done using Analyze 14.0 software (AnalyzeDirect, Overland Park, KS) using manually‐drawn volumes of interest on labeled and unlabeled meshes, and corresponding upper thigh muscles for each animal. Intensity‐based semi‐automatic segmentation was used to analyse mesh volume over time, which was only possible on the labeled mesh due to its greater radiopacity versus surrounding tissue.

### Histology

Implanted material was dissected out at 6 weeks post‐implantation, and freeze‐embedded in optimal cutting temperature media (OCT, Thermo Fisher). Samples were cryosectioned (Leica, Bright 5040) at 15 and 5 µm onto glass slides (Thermo‐Fisher, Superfrost Plus), and left to air dry at room temperature. Tissue was then fixed on the slides in 4% buffered formaldehyde solution for 5 min, before washing in phosphate buffered saline (×3). Staining was done using an automated H and E protocol using a Tissue‐TEK DRS autostainer (Sakura). Neutrophils and Leukocytes were counted manually at 40× magnification from representative slides at each timepoint using a AE2000 microscope (Motic), with five random fields per animal. Digital images were taken at 5 mega‐pixels with an eyepiece camera (Dino‐Eye‐Lite). Collagen staining was done using a Picro Sirius Red kit (Abcam, ab150681) according to the manufacturer's instructions. Representative images of collagen matrixes were taken at 10× magnification, and threshold analysis was done manually using ImageJ's (NIH) measure function to measure % of area coverage.

### Statistical Analysis

All statistical analysis was performed in Graph Pad Prism 6, according to the details in the main text.

## Conflict of Interest

Some of the methods disclosed in his manuscript are covered in a patent application filed at the UK Intellectual Property Office on behalf of PSP and UCL Business.

## Supporting information

Supporting Information

Supplemental Video 1

Supplemental Video 2

Supplemental Video 3

Supplemental Video 4

Supplemental Video 5

Supplemental Video 6

## Data Availability

The data that support the findings of this study are available from the corresponding author upon reasonable request.
