# Supplementary material for: X‐Ray Visible Protein Scaffolds by Bulk Iodination
Source: Adv Sci (Weinh). 2023 Dec 25;11(10):2306246. doi: 10.1002/advs.202306246 (PMC10933627; doi:10.1002/advs.202306246)
Supplement: Supplementary file 1 — Supporting Information [file ADVS-11-2306246-s004.pdf]

ADVANCED SCIENCE
Open Access

Supporting Information

for *Adv. Sci.*, DOI 10.1002/advs.202306246

X-Ray Visible Protein Scaffolds by Bulk Iodination

*Carlos Flechas Becerra, Lady V. Barrios Silva, Ebtehal Ahmed, Joseph C. Bear, Zhiping Feng, David Y.S. Chau, Samuel G. Parker, Steve Halligan, Mark F. Lythgoe, Daniel J. Stuckey and P. Stephen Patrick**

Supplementary information:

X-ray Visible Protein Scaffolds by Bulk Iodination

Carlos Flechas-Becerra[1], Lady Barrios-Silva[2], Ebtehal Ahmed[1], Joseph C. Bear[3], Zhiping Feng[1], David Y.S. Chau[2], Samuel G. Parker[4], Steve Halligan[4], Mark F. Lythgoe[1], Daniel J. Stuckey[1], P. Stephen Patrick[1*]

1. Centre for Advanced Biomedical Imaging, Division of Medicine, University College London, London, WC1E 6DD, UK

2. Division of Biomaterials and Tissue Engineering, Eastman Dental Institute, University College London, Royal Free Hospital, Rowland Hill Street, NW32PF, London, UK

3. School of Life Science, Pharmacy & Chemistry, Kingston University, Penrhyn Road, Kingston upon Thames, KT1 2EE, UK

4. Centre for Medical Imaging, University College London UCL, Charles Bell House, 43-45 Foley Street, London, W1W 7TS, UK

*Corresponding author: peter.patrick@ucl.ac.uk

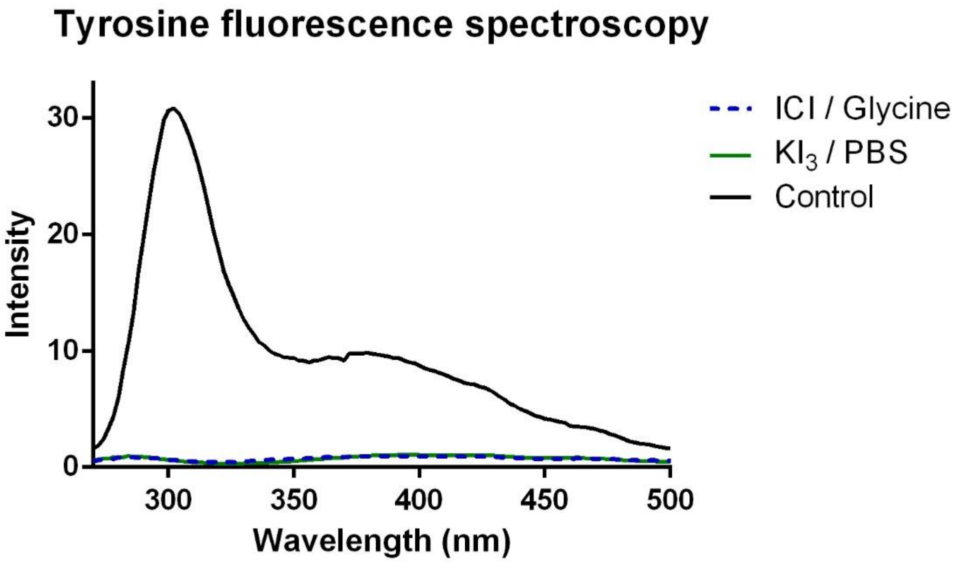

Figure S1. Fluorescence spectroscopy showing decreased tyrosine peak at 300nm following labelling of Xenmatrix decellularised porcine dermis with either the potassium tri-iodide reaction in PBS (pH 7.4), or the iodine monochloride method in glycine buffer (pH 8.6). Samples were excited at 230nm, and emission intensity recorded for 200ms in 2nm steps using a fluorescence spectrometer (Thermo-Fisher Varioskan Lux). Spectra show the average of n=3 independently labelled samples or control unlabeled samples. Intensity at 300 nm was significantly reduced in KI_3 / PBS (0.65 ± 0.25 SD) and ICl / Glycine (0.63 ± 0.07 SD) vs control samples (30.6 ± 1.8 SD) for both labelling methods (2-tailed t-test, $p<0.005$).

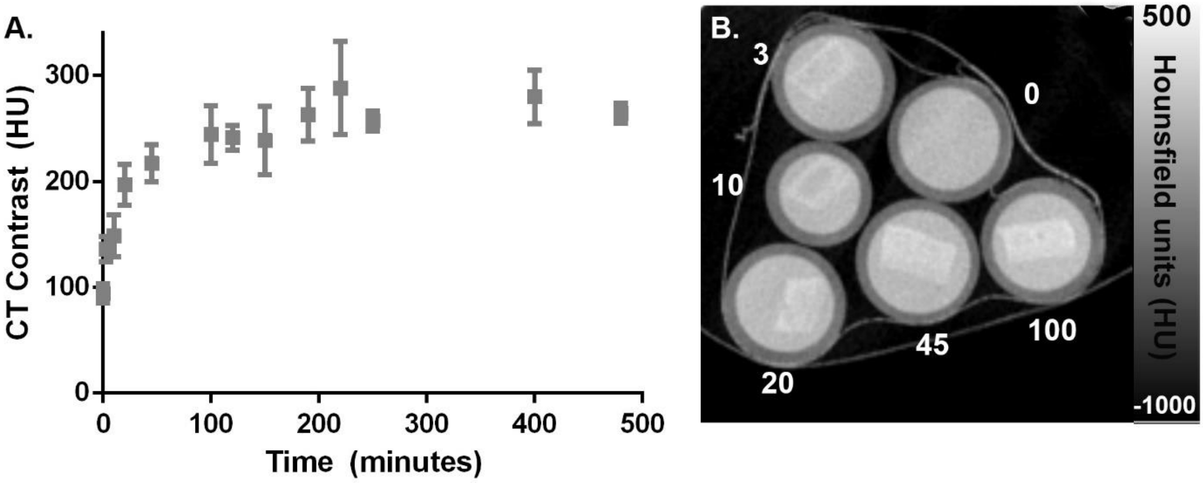

Figure S2. **A.** Reaction kinetics of the KI_3 labelling method in PBS, as measured on decellularised porcine dermis (Xenmatrix) following different incubation times using x-ray CT at 50 kVp. After the indicated reaction times samples were washed in saline for 48 hours to remove unreacted iodine. **B.** X-ray CT image of representative samples from A, at the indicated times in minutes. Surface modification, with low penetration of the reagents to the inside of the mesh can be seen in 3 and 10 minute reaction samples. The 0 minute (unlabelled control sample) is not visible due to its similar radiopacity to the surrounding saline solution. Labelling was found to be rapid, with 80% of the peak radiopacity achieved following a 45 minute reaction period.

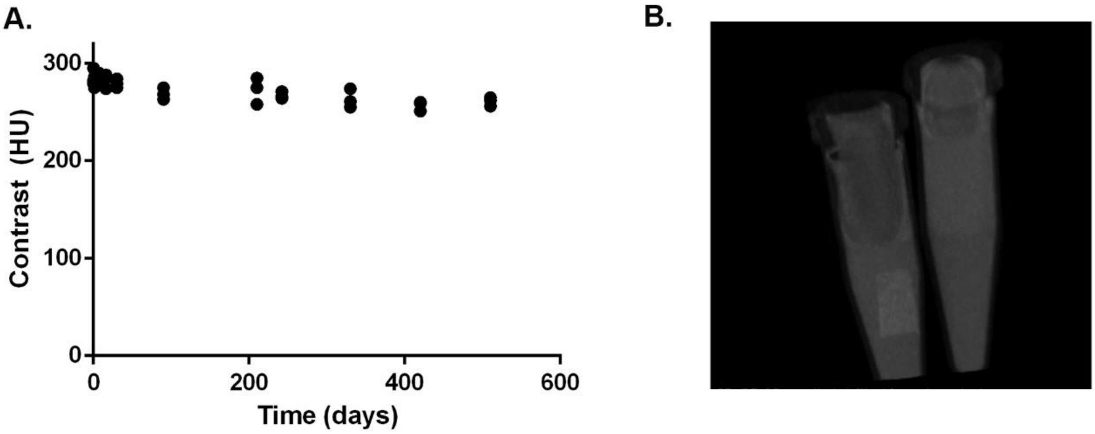

Figure S3. A. Decellularised porcine dermis (Xenmatrix) labelled with the KI_3 / PBS reaction retains its contrast above background over 17 months (510 days), as measured at intervals using X-ray CT at 50 kVp. **B.** Maximum intensity projection of a representative labelled (left) and unlabelled (right) sample of Xenmatrix suspended in saline at 510 days post labelling.

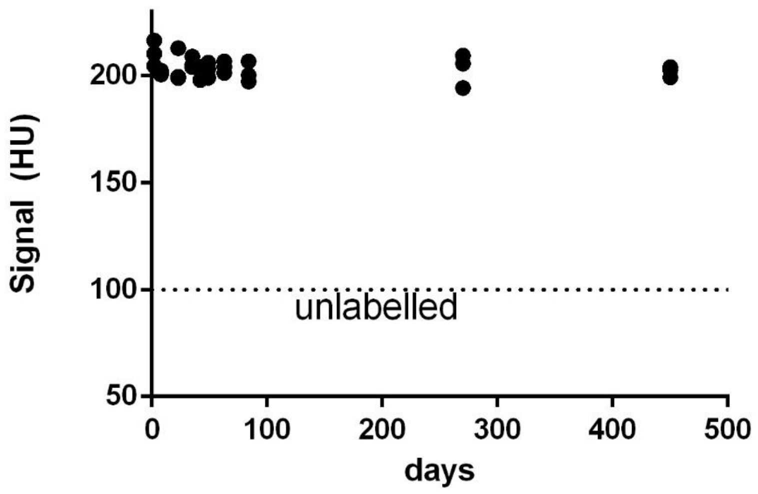

Figure S4. Labelled Neuragen collagen tubes retain their signal over 15 months in saline solution (0.9 % w/v NaCl) at room temperature. Signal was quantified following CT acquisition at 50 kVp and ROI analysis.

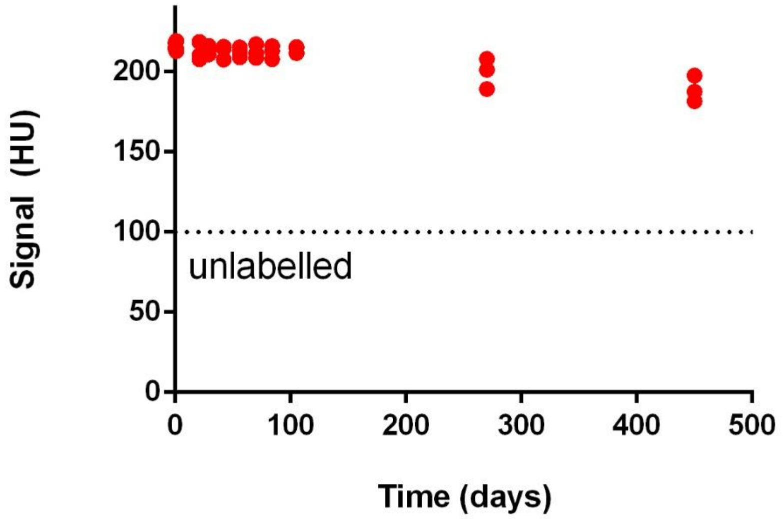

Figure S5. Labelled Egg-shell membrane retain their signal over 15 months in saline solution (0.9 % w/v NaCl) at room temperature. Signal was quantified following CT acquisition at 50 kVp and ROI analysis.

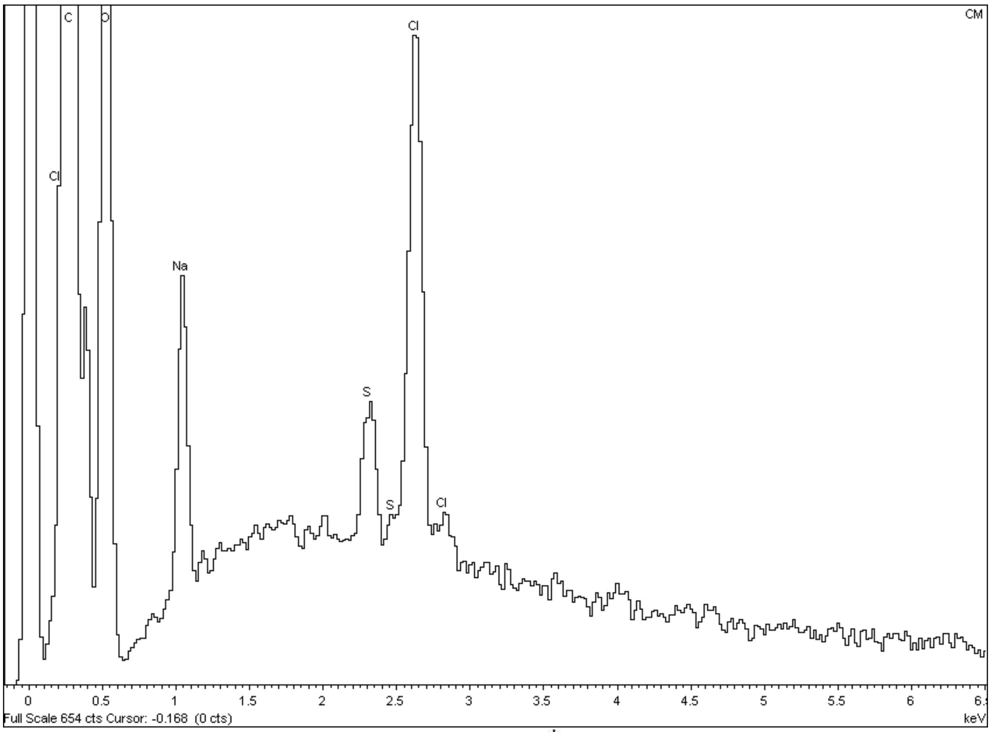

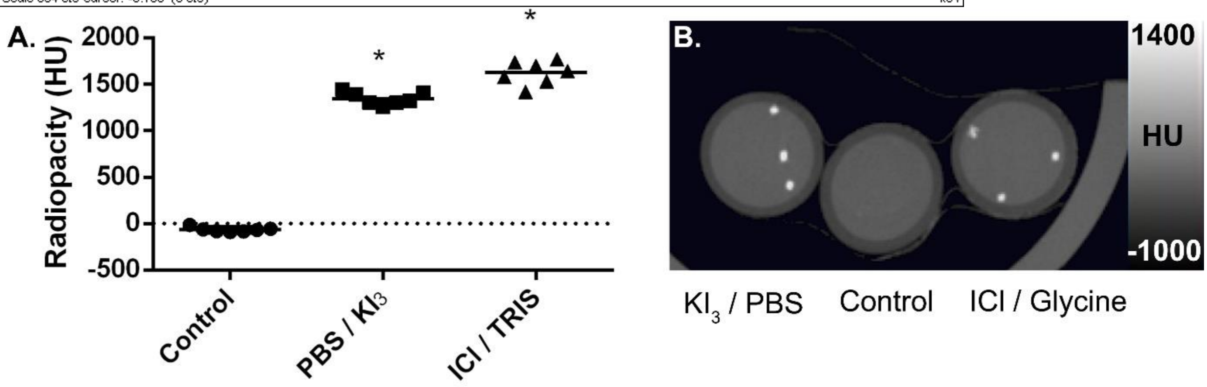

Figure S6 A. Quantification of increased radiopacity of silk fibres (Ethicon Permahand 3-0) post labelling with either indicated reaction. Samples were labelled for 24 hours and washed over 5 days with rotation in saline solution. N=7 shown as individual replicates with mean, *=$p<5x10^{-8}$ (t-test vs control unlabelled). **B.** Representative CT cross section showing visible silk fibres post-labelling, imaged at 90kVp.

Figure S7. Energy Dispersive X-Ray Spectroscopy showing the absence of iodine in stock unmodified mesh (Xenmatrix, Bard).

Figure S8. Energy Dispersive X-Ray Spectroscopy showing the absence of iodine in control (sham) labelled mesh (Xenmatrix, Bard)

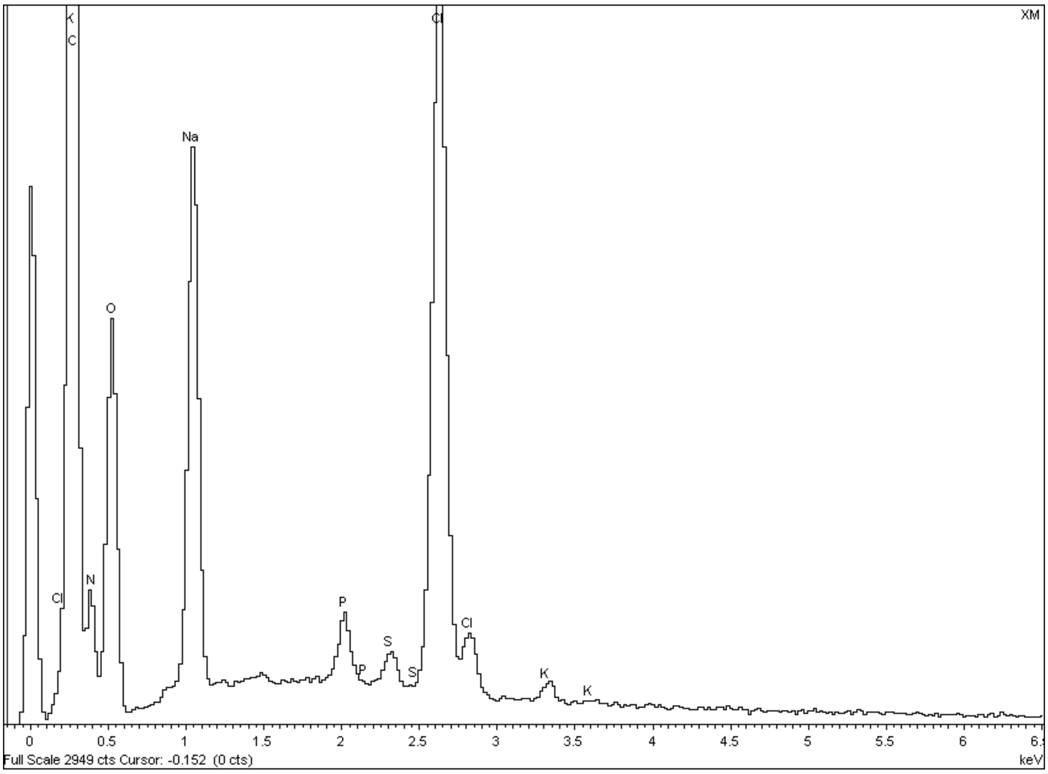

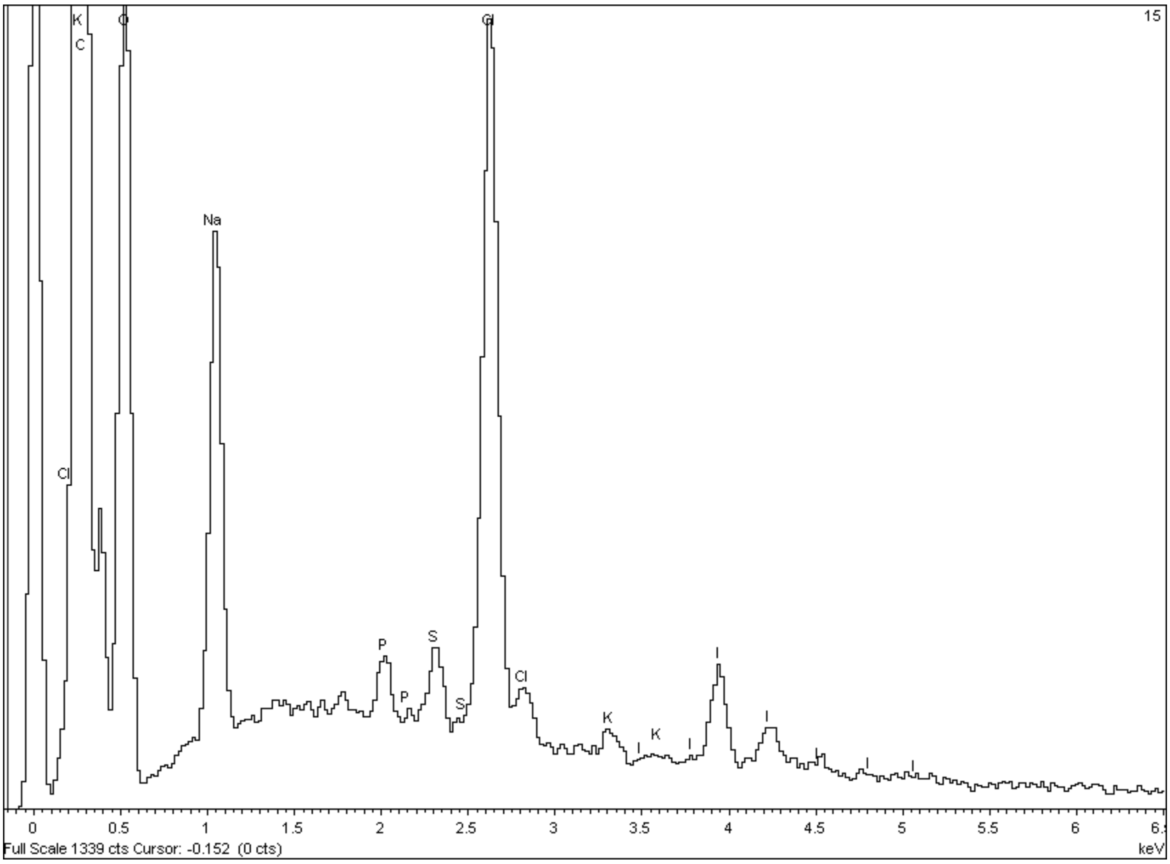

Figure S9. Energy Dispersive X-Ray Spectroscopy showing the presence of iodine in ICl/Glycine labelled mesh (Xenmatrix, Bard).

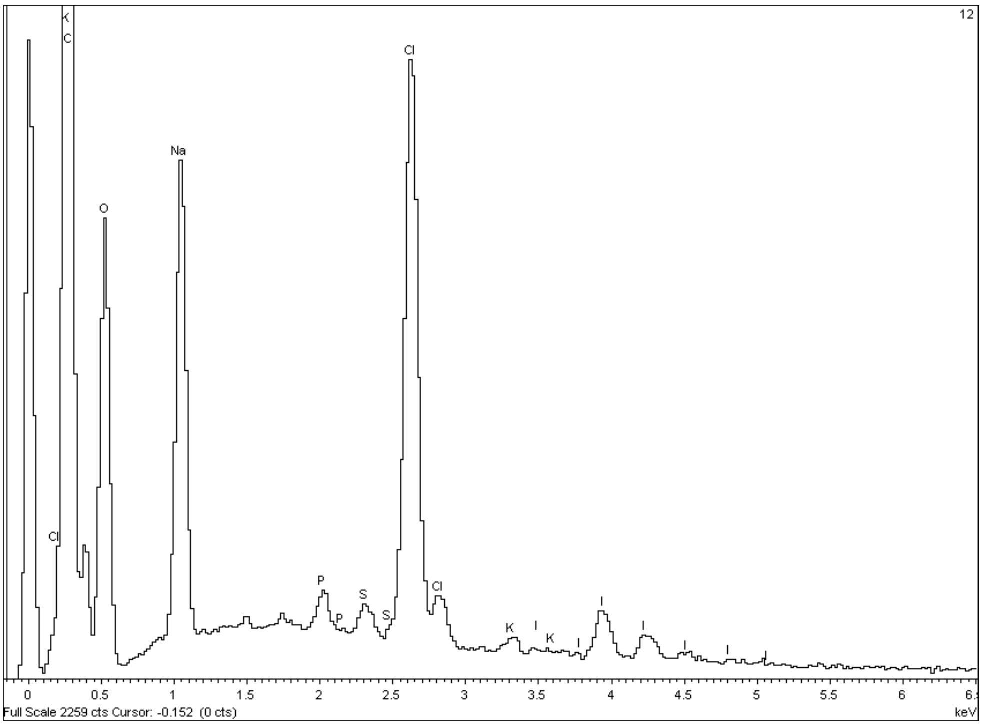

Figure S10. Energy Dispersive X-Ray Spectroscopy showing the presence of iodine in KI_3/PBS labelled mesh (Xenmatrix, Bard).

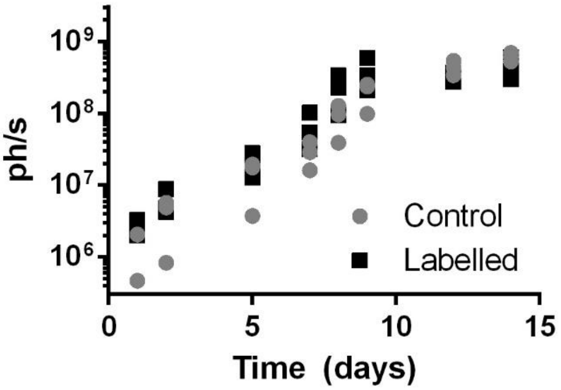

Figure S11. Labelling showed no significant effect on the growth rate of mouse mesenchymal stem cells, with doubling times in the exponential phase of 22.93 hours (±1.03) and 24.38 hours (±3.23 SD) for MSCs on the labelled and unlabelled materials respectively (p=0.49, 2-tailed T-test. ATP metabolism of the cell population was measured non-invasively via transgenic expression of luciferase, with all measurements recorded as photons per second per scaffold at 20 minutes post addition of the substrate luciferin. Points show repeats made on separate scaffold samples seeded with 2000 cells (n=3 per condition). No significant difference was found between samples at any timepoint (p>0.1, 2-tailed T-test).

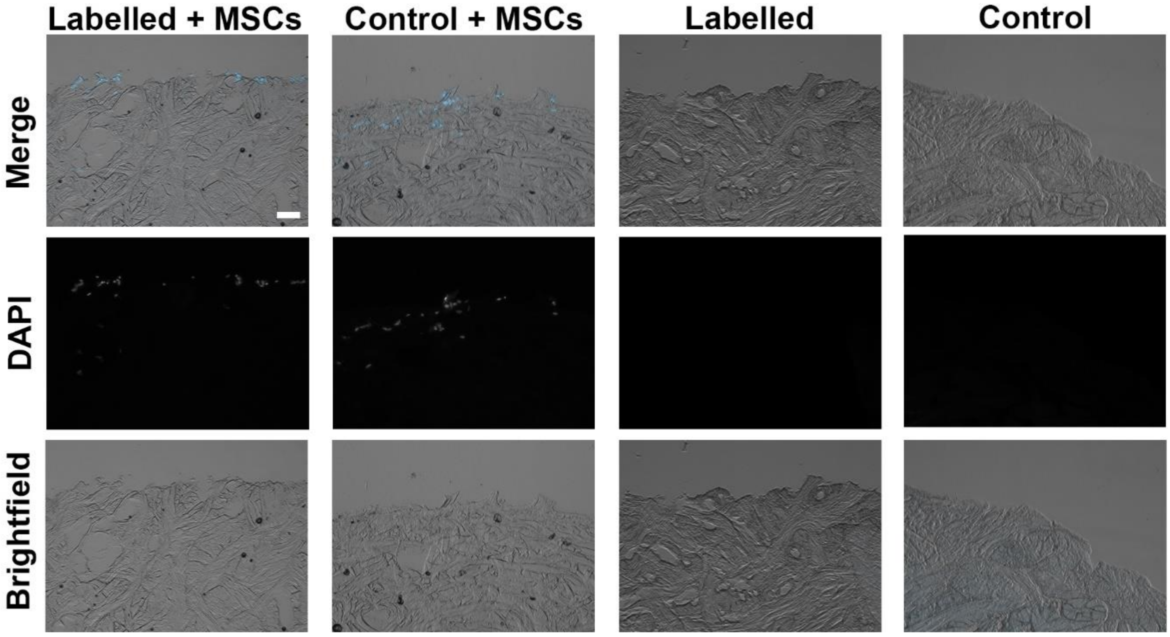

Figure S12. Representative light micrographs following the measurements above (Figure S11), showing the predominantly surface-localised distribution of mouse MSCs at 14 days post seeding on labelled (Lugols/PBS) and unlabelled collagen scaffolds (Xenmatrix). Equivalent unseeded samples show the absence of DAPI-positive nuclei pre-seeding. Scale bar shows 50µm. Samples were cryo-sectioned at 10µm thickness and mounted with Fluoroshield Mounting Medium With DAPI, and imaged using a Zeiss Axiovert 5 fluorescence microscope using a 20x objective.

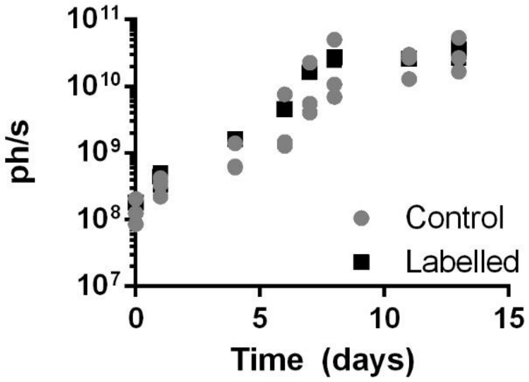

Figure S13 Labelling showed no significant effect on the growth rate of human embryonic kidney cells (HEK 293T), with doubling times in the exponential phase of 24.19 hours (±4.73) and 23.56 hours (±4.69 SD) for cells on the labelled and unlabelled materials respectively (p=0.83, 2-tailed T-test. ATP metabolism of the cell population was measured non-invasively via transgenic expression of luciferase, with all measurements recorded as photons per second per scaffold at 20 minutes post addition of the substrate luciferin. Points show repeats made on separate scaffold samples seeded with 2000 cells (n=3 per condition). No significant difference was found between samples at any timepoint (p>0.05, 2-tailed T-test).

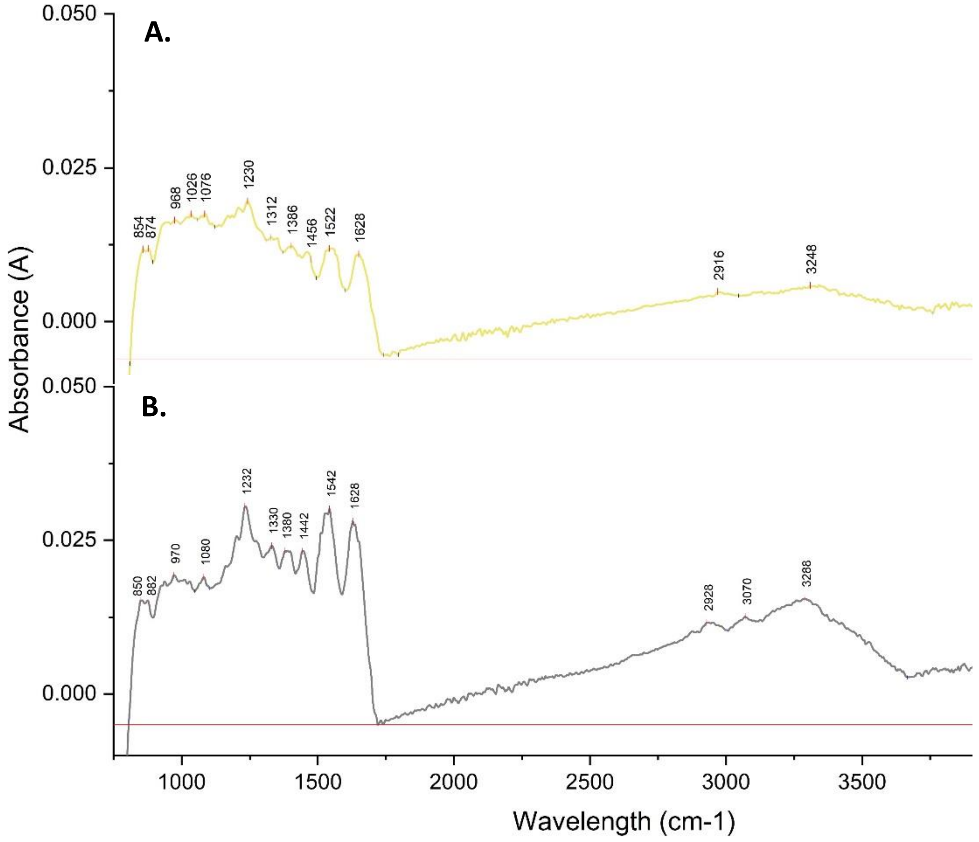

Figure S14. FTIR spectra for **A.** KI_3/PBS labelled mesh, and **B.** control (unmodified) mesh samples.

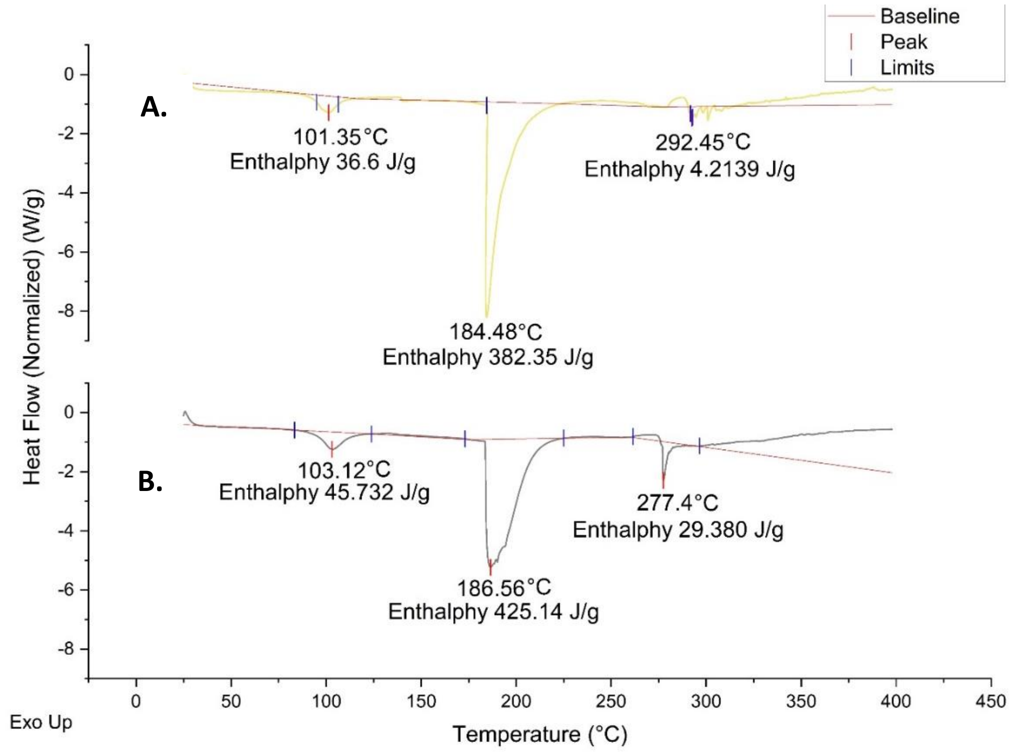

Figure S15. Differential Scanning calorimetry of for **A.** KI_3/PBS labelled mesh, and **B.** control (unmodified) mesh samples cycled from 25°C to 400°C at 10°C/min.

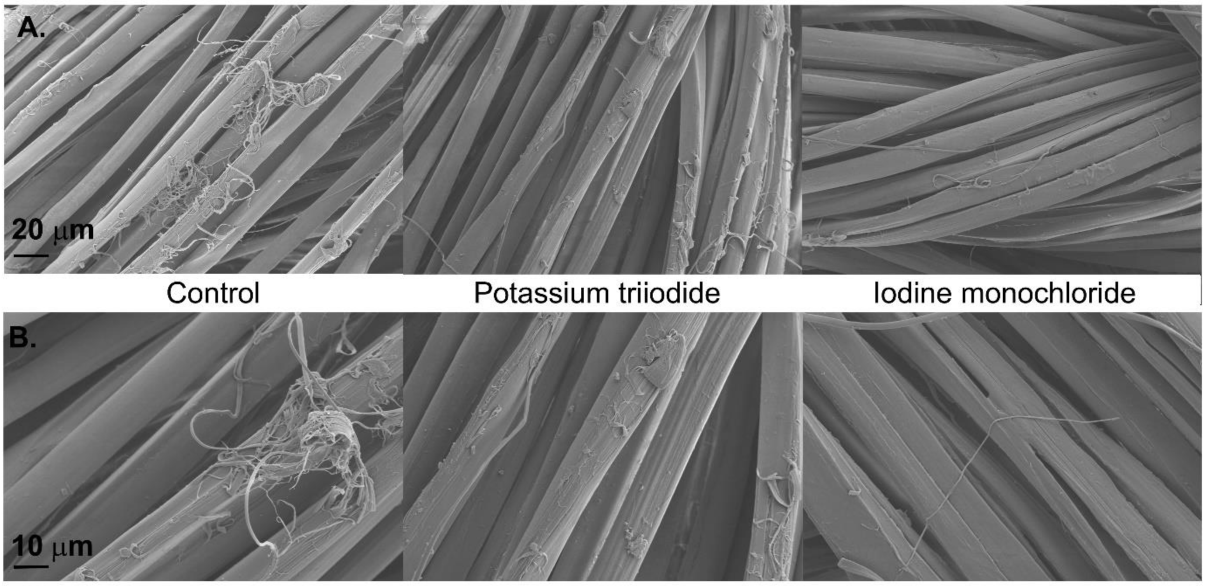

Figure S16. Surface morphology of silk sutures (Ethicon Permahand, 3-0) is comparable between control (unlabelled) samples and those labelled using Potassium triiodide and Iodine monochloride methods (2 day labelling time), as seen with scanning electron micrographs at **A.** low and **B.** high magnification.

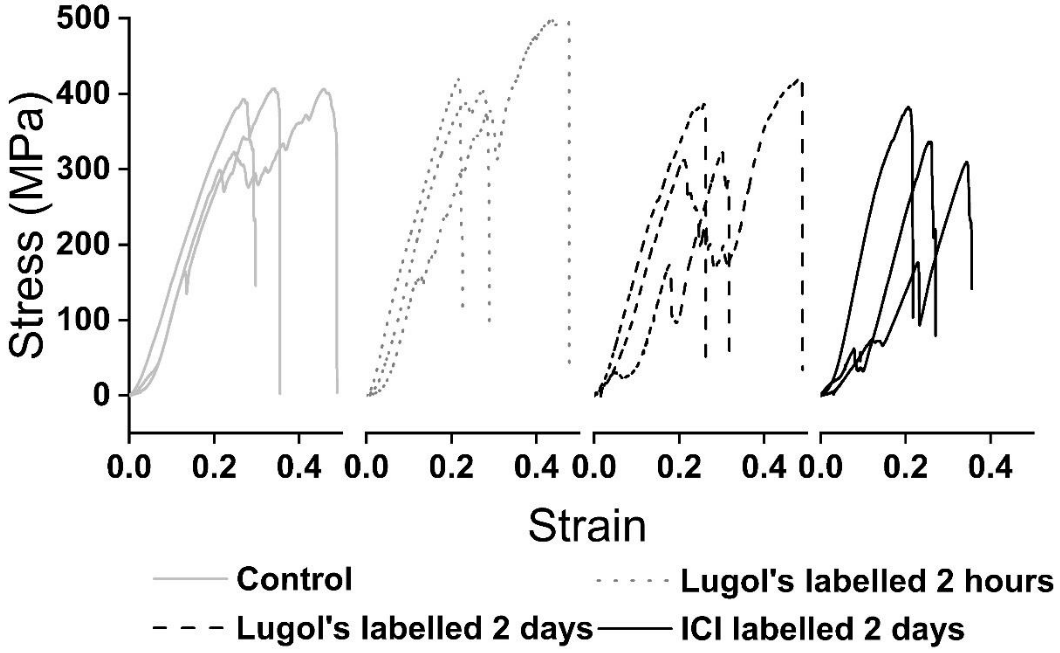

Figure S17. Stress strain curves of silk sutures (Ethicon Permahand 3-0) show no change in mechanical behaviour following labelling with either KI_3 (Lugols' Iodine) or Iodine Monochloride (ICl) reactions, one line per replicate.

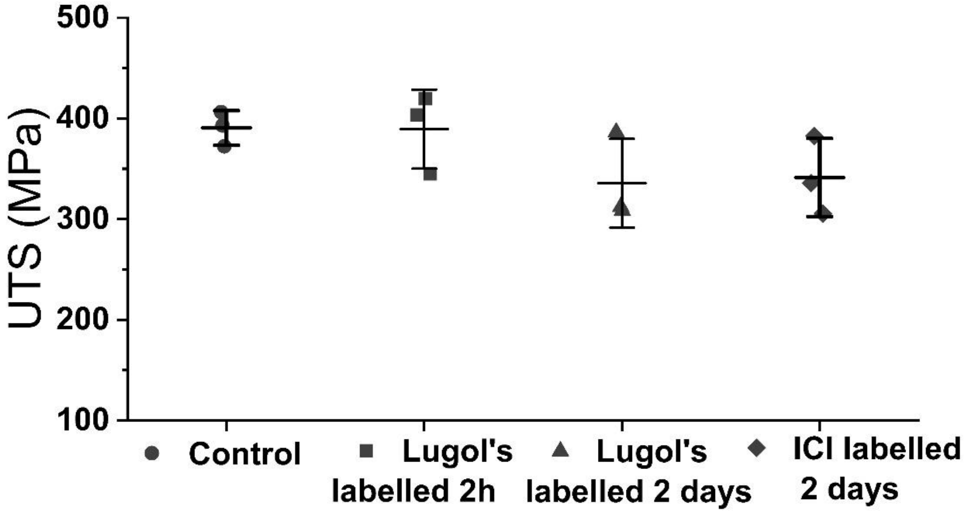

Figure S18. Ultimate tensile strength of silk sutures (Ethicon Permahand 3-0) showed no significant change following labelling with either KI_3 (Lugols' Iodine in PBS with 2 hour or 2 day labelling time) or Iodine Monochloride (ICl in Glycine, 2 day incubation time), (n=3, error bars show SD, p-value>0.05).

**Sample**	**Ultimate tensile strength UTS (MPa)**	**Young Modulus**
**Control**	**390.66±17.13**	**1781.09±147.40**
**KI_3 / PBS (2 hours)**	**389.45±39.25**	**1984.76±414.78**
**KI_3 / PBS (2 days)**	**335.61±44.06**	**1891.55±127.54**
**ICl / Glycine (2 days)**	**341.3±38.91**	**1556.28±1156.99**

Table S1. No significant change was found in the UTS and Youngs modulus of silk sutures (Ethicon Permahand, 3-0) after labeling with KI_3 or ICl reactions, as compared to control (unmodified) samples. Values are presented as means with SD, p>0.05 ANOVA.

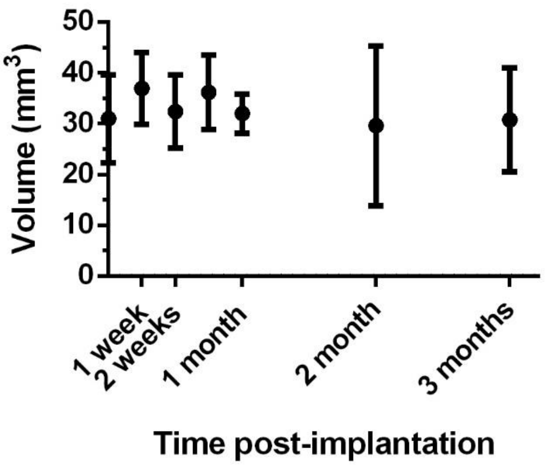

Figure S19. Volume of labelled meshes showed no significant trend decrease over time (R^2=0.0186), as obtained *via* CT-based semi-automatic region of interest segmentation (n≥5).
